# The history of transcatheter aortic valve implantation: The role and contribution of an early believer and adopter, the Netherlands

**DOI:** 10.1007/s12471-020-01468-0

**Published:** 2020-08-11

**Authors:** P. de Jaegere, M. de Ronde, P. den Heijer, A. Weger, J. Baan

**Affiliations:** 1 Department of Cardiology, Erasmus University, Rotterdam, The Netherlands; 2 Department of Cardiology, Amphia Hospital, Breda, The Netherlands; 3 Department of Cardiothoracic Surgery, Leiden University Medical Centre, Leiden, The Netherlands; 4 Department of Cardiology, Amsterdam AMC, University of Amsterdam, Amsterdam, The Netherlands

**Keywords:** Aortic stenosis, TAVI

## Abstract

This paper describes the history of transcatheter aortic valve implantation (TAVI) from its preclinical phase during which visionary pioneers developed its concept and prototype valves against strong head wind to first application in clinical practice (2002) and the clinical and scientific role of an early believer and adopter, the Netherlands (2005).

## Dutch contribution to the field

- First TAVI via the axillary artery, June 2006.
- First true percutaneous TAVI, October 2006.
- Contribution to TAVI using local anaesthesia.
- Cerebral protection.

## Introduction

2020 is the year that the Netherlands was to host the annual meeting of the European Society of Cardiology (ESC) whose mission is to reduce the burden of cardiovascular disease through education, congresses, surveys and publishing [1]. We as medical professionals as well as those who are directly or indirectly involved in the deterrence of illness or ailment and/or the delivering of care (e.g. healthcare authorities such as governments, controlling & advisory bodies, insurance companies, medical industry, etc.), should in addition to that statement also be inspired by the US Food and Drug Administration (FDA) that has taken the role and responsibility of ensuring the timely availability of innovative, safe and effective products to the American people [2].

Transcatheter aortic valve implantation (TAVI) is an example of such a technology that has proven to reduce disease burden by improving quality of life and survival in patients with aortic stenosis [3–9]. Because of its minimally invasive nature (local anaesthesia, minimal incision, beating heart procedure, no cardiopulmonary bypass, …) and its undeniable efficacy as it reduces valve stenosis, it has been embraced by physicians, patients and relatives. This enthusiasm is supported by the findings showing overall clinical equivalence between TAVI and surgical aortic valve replacement (SAVR) which necessitates general anaesthesia, extensive surgical trauma, cardiac arrest and cardiopulmonary bypass. TAVI is a disruptive technology and has caused a sea change in cardiovascular therapy similar to intracoronary stenting 40 years earlier [10].

At the cradle of TAVI are the visionary pioneers in Europe who came up with the idea and had the courage to introduce TAVI in clinical practice notwithstanding endless pessimism and even open opposition. Interestingly, the discussion of added clinical value and, thus, appropriateness of reimbursement is still present notwithstanding the consistent findings of the various randomised controlled trials and numerous multicentre surveys. This contrasts with the position of the FDA that has granted approval of TAVI in patients with aortic stenosis at low risk (August 2019). It also contrasts with the respected position of the Netherlands, which ranks fourth on the Global Innovation Index 2019 (after USA, Switzerland, and Sweden) and has been in the top ten of all countries in the world for many years [11]. It also ranks very highly in matters of social, economic and political stability, infrastructure and organisation and belongs to the elected group of high-income countries.

## The early 1990s—Henning Andersen, Aarhus, Denmark

**Fig. 1:**
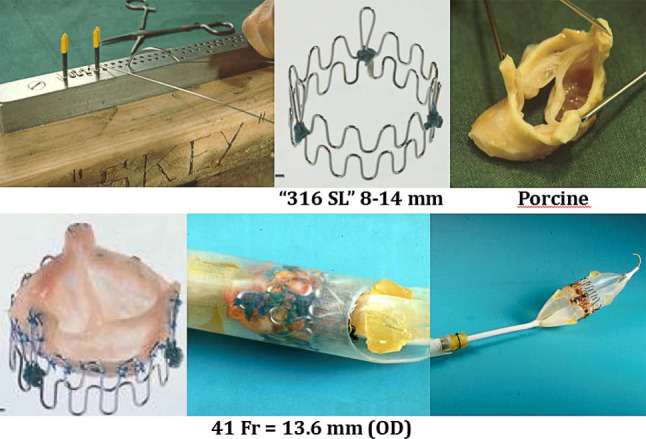
Henning Andersen’s prototype percutaneous aortic valve technology

The TAVI story started in February 1989 when Henning Andersen—inspired by a lecture of Julio Palmaz on the development of coronary stents—thought of inserting a biological valve inside a large stent and to implant this using a balloon-expandable technique similar to the stent technique described by Palmaz. Andersen manufactured a stent himself with a diameter of 30 mm using metal wires that he bought in a hardware store in which he mounted a porcine aortic valve that was crimped onto a second-hand 30 mm balloon catheter pioneered by Cribier in the 1980s. The assembly was then inserted into a 41 Fr. introducer sheath (Fig. 1).

The first implantation on 1 May 1989 in an 80 kg pig was uneventful. Yet, during subsequent experiments coronary occlusion and valve embolisation occasionally occurred. He also found that arresting blood flow prevented balloon migration for which he developed a custom-made balloon-tipped catheter inflated in the common pulmonary trunk.

He presented his work on 19 May 1990 at the Danish Society of Cardiology (Odense, Denmark) but the abstract submitted to the 12th Congress of the ESC in 1990 (Stockholm, Sweden) was rejected. This also held for the paper submitted to the Journal of the American College of Cardiology (1990) and Circulation (1991). Both journals considered ‘it too low a priority for publication’. The paper was ultimately accepted by the European Heart Journal in 1992 (impact factor 1.6) followed by another publication in 1993 [12, 13]. Posters at the ESC and AHA meetings in 1992 received little attention.

Andersen realised that after 41 implantations and the submission of a patent (1993) he needed support from industry, engineering and funding to move forward. Yet, none showed interest as their medical advisers predominantly consisting of cardiothoracic surgeons provided numerous reasons why this could not work. As he could no longer afford the patent-related costs, he sought and received support from Stanford Surgical Technologies (SST), a small company founded by cardiothoracic surgeons in San Francisco, which licensed his patent with the promise to develop the technology. Yet, nothing happened. SST became Heartport and concentrated on the development of a less invasive SAVR (port access) while holding the exclusive license agreement. On 21 January 2001, Heartport was acquired by Johnson&Johnson-IS (JJ-IS) but three days earlier (18 January 2001) Heartport had sold the exclusive license agreement to Percutaneous Valve Technologies (PVT).

## The balloon-expandable valve story—Alain Cribier, Martin Leon, Stan Rabinovich, Stanton Rowe (PVT)

**Fig. 2:**
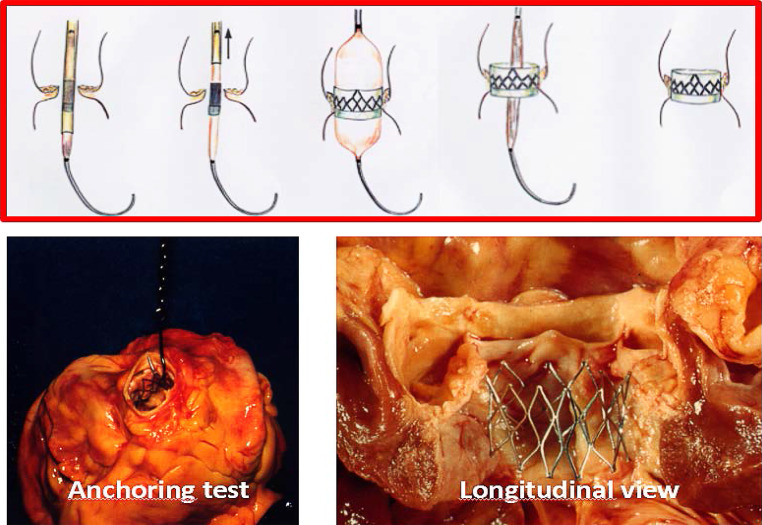
Alain Cribier’s TAVI concept and cadaver experiment. Anchoring test concerns an in vitro test evaluating the stability of the valve that was deployed by balloon expansion within the aortic annulus by suspending the heart after valve implantation

In the mid-1990s Alain Cribier pioneered aortic balloon valvuloplasty (1985) but, confronted with the high restenosis rate, he presented a similar idea to a number of companies (Fig. 2). He knew that a balloon was capable of disrupting a stenotic aortic valve and decided to take advantage of the calcification for frame-anchoring. The first cadaver experiment was performed in 1994. The stent was conceptualised together with a cardiac surgeon (Dr Bessou) in such a way that in its crimped configuration (8 mm) it would be possible to deliver it via the femoral artery. Analogous to Andersen’s experience, companies were not interested as it was considered: ‘*ridiculous, impossible and unnecessary*’. Yet, Stanton Rowe championed the concept at JJ-IS, which licensed Cribier’s ideas and agreed to develop the percutaneous valve. Unfortunately, JJ-IS was at that time (1996) in the midst of acquiring Cordis and nothing happened. Cribier returned to Stanton Rowe and Stan Rabinovich who had both left JJ-IS. They brought Cribier’s idea back to Martin Leon which resulted in the creation of PVT (21 July 1999). In search of venture capital, they came into contact with an Israeli company ARAN R&D (June 1999, Jerusalem) who were interested in investing money but also in the development of the valve. Yet, the development of the percutaneous valve necessitated Andersen’s patents licensed to SST as they contained the fundamentals around a collapsible and expandable valve for which the PVT series A financing was used (December 2000). Despite negative advice from cardiothoracic surgeons, Medtronic and Boston Scientific subsequently became the main investors. A meeting between PVT and Edwards Lifesciences (TCT, September 2003) led to the acquisition of PVT (12 December 2003) after consent from Medtronic and Boston Scientific [14].

The first animal (non-atherosclerotic) experiments using polymeric valves were performed in August 2000 but without success since there was no anchoring. The choice of healthy animals is understandable but surprising given Cribier’s initial experiments with cadaver hearts. Noteworthy is the short time between the animal experiments and the first clinical TAVI (16 April 2002). Cribier was faced with a 57-year-old man in heart failure and a poor left ventricular ejection fraction (10%). To complicate matters, the patient had a failed aorto-bifemoral graft precluding a transfemoral approach for which the valve system was designed. At the risk of jeopardising all the work done, its future and the company the decision was taken to use the valve system via an ‘unplanned’ antegrade-transseptal route given the patient’s fate if nothing was done. In a subsequent feasibility study (36 patients, 2002–2005) the ‘success rate’ was 75% but paravalvular aortic regurgitation frequently occurred since only one size (23 mm) was available. During this study, the value of rapid ventricular pacing for valve delivery was recognised. Now, so many years after this pioneering work, Cribier says *‘… it is moving to remember the fierce opposition of experts towards this “totally unrealistic and stupid idea” that “would never work”’*.

## The self-expanding valve story—Georg Boertlein, Rob Michiels, Jacques Sequin (CoreValve)

CoreValve was founded by a cardiothoracic surgeon Jacques Seguin in Paris in 2001 together with a bio-medical engineer Georg Boertlein, who both understood the future of a catheter-based minimally invasive aortic valve treatment. In 2001, they found Rob Michiels (managing director of CONSILIUM associates active in identification, funding and green-housing of start-up technologies) immediately interested, which led to the entire high risk ‘seed round’ of CoreValve (mid-2002) and paved the way to the first-in-man in 2004 despite the fact that *‘… well-regarded medical professionals told them that we were crazy and it would be over their dead body if one of these ever got implanted in a patient*’.

The CoreValve technology featured a novel leaflet and construction design using porcine pericardium to allow for more compression capability, thereby reducing catheter size and developing a true ‘interventional’ device. CoreValve chose a strategy of restricted use by a small number of centres in Europe that was continued after CE marking in 2007 for the 3th generation to assure successful maturing of their technology, appropriate training of physicians and to gather additional clinical data for post-approval surveillance later submission to the FDA.

## TAVI in the Netherlands

**Fig. 3:**
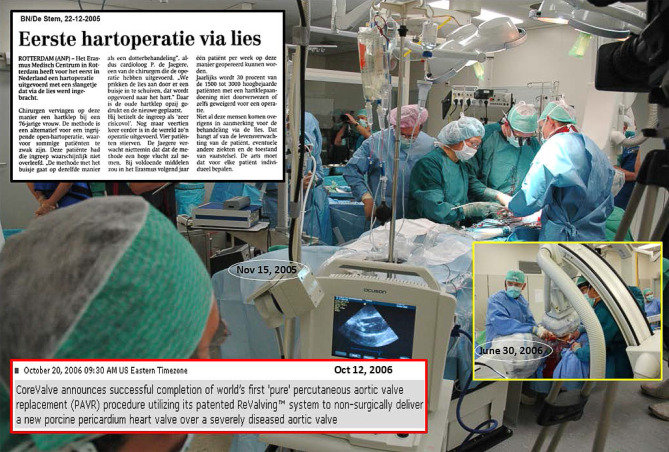
First TAVI in the Netherlands in 2015 and milestones in 2006. Professor Serruys, Dr Kappetein and Dr de Jaegere during the first TAVI in the Netherlands on 15 November 2005. General anaesthesia, surgical cut-down, ECMO, CoreValve 26 mm valve. The patient died 12 years later (2017). *Inset upper left*: Dutch newspaper reporting ‘First heart operation via groin’. *Inset lower right*: first TAVI via the subclavian artery (30 June 2006), *inset lower left*: CoreValve press release on 20 October 2006 reporting first full percutaneous TAVI in the world on 12 October 2006

**Fig. 4:**
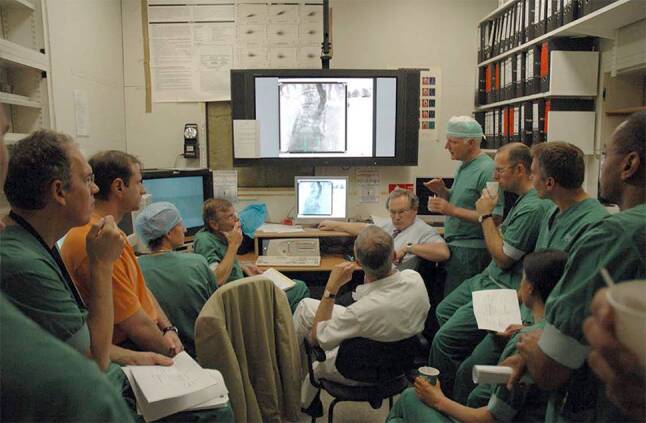
Briefing before TAVI. Briefing before the first TAVI via the subclavian artery (CoreVale 26 mm). Seating in front from *right to left*: M de Ronde (head nurse), Dr de Jaegere, Dr Kappetein (white coat, back), Professor Serruys. Standing behind Professor Serruys: Dr Klein (anaesthesiologist). Please note the ‘script’ in the hands of attendees summarising all procedural steps and materials that were needed in chronological order during the planned procedure

The first TAVI in the Netherlands was performed in the Erasmus Medical Center, Rotterdam on 15 November 2005 using the self-expanding CoreValve (Fig. 3). [15] The team first conducted a short animal experiment to get a feel for the catheter system and technique of delivery (September 2005). The product was not CE-marked and, therefore, permission for compassionate use was granted by the Ministry of Health. Given the experimental nature and limited experience, a script was written in which all steps in chronological order were summarised including the materials and responsibility of each team member throughout the procedure (Fig. 4). This was continued during the early years of TAVI (2005–2007), which established a disciplined surgical-type approach in the intervention room that became an undisputable and natural modus operandi. Shortly after this, and in close cooperation with the Erasmus Medical Center, the second TAVI and first inclusion in the CoreValve first-in-man study was performed in the Amphia Hospital, Breda in February 2006 (Tab. 1).

**Table 1:** Summary start TAVI in the Netherlands

	Year	Month	Day	Hospital	City
1	2005	11	15	Erasmus MC	Rotterdam
2	2006	2	9	Amphia	Breda
3	2007	6	8	St Antonius	Nieuwegein
4	2007	10	1	AMC	Amsterdam
5	2007	11	8	LUMC	Leiden
6	2008	4	10	UMCU	Utrecht
7	2008	11	26	MCL	Leeuwarden
8	2008	–	–	Catharina	Eindhoven
9	2008	–	–	Radboud	Nijmegen
10	2008	–	–	UMCM	Maastricht
11	2009	5	27	UMCG	Groningen
12	2009	10	15	OLVG	Amsterdam
13	2009	12	2	Isala	Zwolle
14	2012	11	13	MST	Enschede
15	2013	8	27	Haga	The Hague
16	2014	10	30	VUMC	Amsterdam

Population of the Netherlands (2018): 17.2 million (population density of 488 people/km^2^)

Gross domestic product/capita (2018): 53,228$

Hospital beds/1000 people: 5.8 (1990)–4.7 (2009). (USA 3.1 in 2009—source WHO)

At that time, some sort of circulatory support was recommended. In the first and the second patient (4 April 2006) an extracorporeal membrane oxygenation system was used but replaced by the TandemHeart in the next three as this allowed percutaneous insertion. It was also the period that an interventional radiologist experienced with percutaneous endovascular aortic repair (Lucas van Dijk) trained the team in echo-guided arterial access. This in combination with the use of a percutaneous closure device (Prostar) and the fact it turned out that TAVI could be performed without circulatory support (stable haemodynamics when reducing flow) led to the first fully percutaneous TAVI in the world (12 October 2006, Fig. 3). [16] A milestone that has been adopted world-wide and has become the standard for transfemoral TAVI (TF-TAVI). Of note, this was preceded by another first-in-the-world, namely TAVI via the subclavian artery on 30 June 2006 (Fig. 4), which has become the dominant approach in Radboud University Medical Center.

**Fig. 5:**
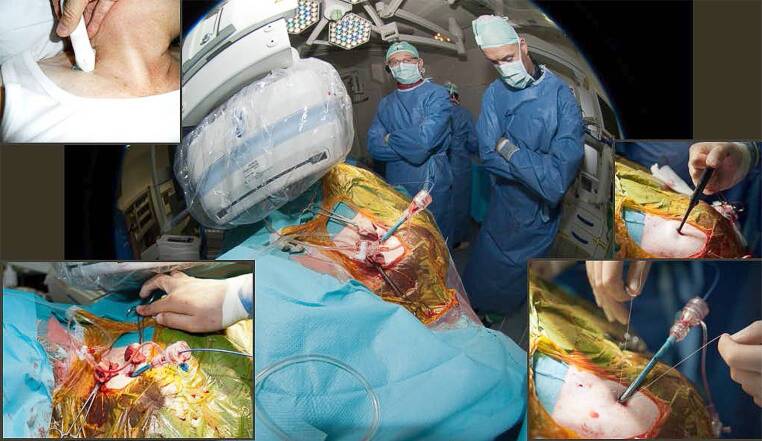
TAVI via the axillary artery under local anaesthesia. Procedure (Medtronic CoreValve 31 mm) performed by Dr van Mieghem and Dr de Jaegere on 13 September 2011. Echo-guided access followed by application of suture-based closure system, valve implantation and percutaneous closure

The next major step was the use of local anaesthesia. This was pioneered in the Netherlands by the team at the Academic Medical Center Amsterdam and first performed in 2010. It was the stepping stone for further simplification of TAVI to a minimalist approach mimicking PCI [17]. Moreover, TF-TAVI is now possible via a two-arterial access only (femoral artery for valve delivery and pacing over the wire, contralateral femoral or radial for pig-tale guidance) [18]. In case of TAVI for failed bioprosthesis, single access (femoral) suffices as the radiopaque structures of the bioprosthesis can be used as reference for valve deployment. During all those innovations, a fully percutaneous TAVI via the axillary artery under local anaesthesia became a reality and was first performed on 13 September 2011 (Fig. 5). In conjunction with the experience gained and improved catheter and valve technology, a program of early discharge was instituted [18–21]. The Netherlands also played an important role in the adoption and evaluation of the use of cerebral protection devices for the prevention of perioperative stroke [22]. Last but not least and perhaps more importantly, the typical Dutch spirit of consultation and collaboration has led to structured multidisciplinary decision-making, planning, execution and evaluation involving medical specialists with various backgrounds and expertise ensuring balanced treatment stratification via the heart-team [23]. Given the outstanding infrastructure in the Netherlands, such as the nationwide prospective registry that was created under the auspices of the Netherlands Society of Cardiology and Cardio-Thoracic Surgery to improve quality of care by monitoring patient demographics and clinical outcomes (BHN-registratie), clinical programs are incorporated into clinical-scientific ones [24]. The TAVI Care and Cure is an example of this [25]. The clinical scientific output of the Netherlands is summarised in Tab. 2 and 3. Beyond the analysis of outcomes and the underlying mechanisms in single, multicentre national and international initiatives and collaborations, research has been initiated to elucidate and predict the interaction between the device and host as well as the role of Artificial Intelligence in TAVI [26–35]. The clinical drive of innovation providing the best possible care to the individual patient and the scientific work (volume and content) of all Dutch medical professionals is an expression of the stimulating environment in which they have the pleasure to live and work.

**Table 2:** Peer-reviewed papers (source PubMED, EndNote X9) by Dutch investigators (as of 1 March 2020)

Year	*n*	≥15	≥10–15	0–10
2007	1	0		1
2008	3			3
2009	1			1
2010	15	1	1	13
2011	13			
2012	18	2	1	15
2013	22	2	3	17
2014	36	5		
2015	30	2	1	27
2016	33	2	1	30
2017	48	9	0	39
2018	48	6	0	42
2019	54	2	0	52
2020	24	2	0	22

Total number of publications (*n*) per year are subdivided by the journal Impact Factor (2019) using the following categories: ≥15, 10–15 and 0–10

**Table 3:** PhD theses by Dutch Academic Institutions

	Year	Institute	1th Promotor	1th Copromotor	Candidate	Title
1	2011	EMC	Serruys	De Jaegere	Tzikas	The role of advanced imaging in TAVI
2	2011	EMC	Serruys	De Jaegere	Piazza	TAVI: from experiment to clinical practice and beyond
3	2012	AMC	Piek	Baan	Yong	Clinical and haemodynamic effects of TAVI
4	2013	EMC	De Jaegere	Van Domburg	Nuis	TAVI: Current results, insights & future challenges
5	2014	AMC	Piek	Baan	Van Dijk	Percutaneous treatment of heart valve disease
6	2014	EMC	De Jaegere	n.a.	Van Mieghem	Transcatheter aortic valve therapies: from cutting edge to mainstream
7	2014	EMC	De Jaegere	Van Domburg	Van der Boon	Insights into complications of TAVI
8	2014	LUMC	Bax	Delgado	Katsanos	Outcomes of TAVI
9	2014	UMCM	Prinzen	Van Gelder	Houthuizen	Left bundle branch block: controversies in aortic interventions and cardiac resynchronisation therapy
10	2014	UMCU	Doevendans	Stella	Samim	TAVI; optimisation of the technique, assessment of complications an future directions
11	2015	UMCU	Doevendans	Stella	Nijhoff	Evolving concepts in TAVI
12	2016	AMC	Piek	Baan	Wiegerinck	Replacing the valve, restoring flow. Effects of TAVI
13	2016	AMC	Van Bavel	Marquering	Elattar	Quantitative image analysis for planning of aortic valve replacement
14	2016	LUMC	Bax	Delgado	Ewe	Aortic valve disease: novel imaging insights from diagnosis to therapy
15	2018	AMC	Piek	Baan	Kesteren van	Screening complications and outcome of aortic valve implantation
16	2018	EMC	De Jaegere	Van Mieghem	Gils van	TAVI: insights and solutions for clinical complications and future perspectives
17	2018	UMCM	Prinzen	Houthuizen	Poels	Left bundle branch block in TAVI
18	2019	AMC	Piek	Delewi	Vlastra	Cerebral outcomes in patients undergoing TAVI
19	2019	AMC	De Winter	Tijssen	Abdelghani	Transcatheter interventions for structural heart disease: present and future
20	2019	AMC	Henriques	Vis	Van Mourik	Percutaneous treatment of aortic valve disease- Towards optimal patient outcomes
21	2019	EMC	Kappetein	Piazza	Mylotte	Evolution of transcatheter heart valve technology
22	2020	UMCU	Doevendans	Stella	Kooistra	Individualised optimisation of TAVI
23	2020	UMCU	Doevendans	Stella	Abawi	Role of novel predictive factors on clinical outcome after transcatheter aortic valve replacement
24	2020	EMC	Mattace Raso	Lenzen	Goudzwaard	The impact of frailty on outcome after TAVI in older patients
25	2020	EMC	De Jaegere	Lenzen	Faquir	Clinical application of patient-specific computer simulation and advanced imaging in TAVI

https://www.narcis.nl/search/coll/publication/Language/NL/uquery/TAVI
